# The Role of the Endometrial Microbiota in Endometrial Cancer: A Systematic Review of the Literature

**DOI:** 10.3390/jcm13237135

**Published:** 2024-11-25

**Authors:** Guglielmo Stabile, Alessandra Doria, Matteo Bruno, Marco D’Indinosante, Valerio Gallotta, Francesco Fanfani, Giovanni Scambia, Stefano Restaino, Giuseppe Vizzielli, Stefania Carlucci, Luigi Nappi

**Affiliations:** 1 Department of Medical and Surgical Sciences, Institute of Obstetrics and Gynecology, University of Foggia, 71121 Foggia, Italy; guglielmost@gmail.com (G.S.); a.doria3108@gmail.com (A.D.); s.carlucci86@gmail.com (S.C.); luigi.nappi@unifg.it (L.N.); 2 UOC Ginecologia Oncologica, Dipartimento per le Scienze della Salute della Donna e del Bambino e di Sanità Pubblica, Fondazione Policlinico Universitario Agostino Gemelli IRCCS, 00168 Rome, Italy; brunomatteo2@gmail.com (M.B.); valerio.gallotta@policlinicogemelli.it (V.G.); francesco.fanfani@policlinicogemelli.it (F.F.); giovanni.scambia@policlinicogemelli.it (G.S.); 3 Università Cattolica del Sacro Cuore, 00168 Rome, Italy; 4 Department of Medicinal Area (DAME) Clinic of Obstetrics and Gynecology, Santa Maria della Misericordia Hospital, Azienda Sanitaria Universitaria Friuli Centrale, 33100 Udine, Italy; restaino.stefano@gmail.com; 5 Obstetrics and Gynecology Clinic, Department of Medicine, University of Udine, 33100 Udine, Italy; giuseppevizzielli@yahoo.it

**Keywords:** endometrial cancer, endometrial microbiota, microbiome, oncology, uterus

## Abstract

**Background**: Endometrial cancer is currently the sixth most frequent cancer in women, and scientific research is focusing on the search for particular features of the endometrium that may explain a further predisposition to the onset of endometrial cancer, aimed at improving knowledge of the pathogenetic factors of this disease. The aim of our review is to analyze in detail the results of the literature on the endometrial microbiota in patients with endometrial cancer and to investigate its role. **Methods**: We performed our research on the Pubmed, Web of Science, and Scopus databases. We searched up to December 2023 and considered manuscripts published from 2000. Only articles in English were included in the search. We excluded studies in which the endometrial microbiota were collected through the vagina or cervical canal. **Results**: We included in our review a total of five manuscripts at the end of the screening process, and the total number of patients involved was 190. Four studies considered only post-menopausal patients, while one study considered both pre- and post-menopausal patients. In all studies, the microbiota analysis was derived from a post-hysterectomy biopsy. From our review, it emerged that Bacteroidetes, Actinobacteria, Firmicutes, and Proteobacteria are the most represented bacteria in patients with endometrial cancer. These are both Gram-positive and Gram-negative, but predominantly anaerobic bacteria. **Conclusions**: The reduced microbial diversity and the presence of specific bacteria is often associated with endometrial cancer. Further work on larger population samples, and on healthy women and those affected by endometrial carcinoma, is needed to understand how the endometrial microbiota changes and influences the development of the tumor and whether intervening in the changes in the microbiota will have a therapeutic impact on endometrial carcinoma.

## 1. Introduction

Endometrial cancer (EC) is currently the sixth most diagnosed cancer in women, with an incidence of 417,000 new cases in 2020 and around 97,000 deaths [1]. There is a tendency for it to increase, especially in high-income countries, where it represents the most typical gynecological tumor. This increase seems to be linked to the aging of the population and the reduction in hysterectomies for the treatment of benign pathologies [2]. In Italy, endometrial cancer ranks as the third most frequently diagnosed cancer in post-menopausal women, with approximately 8300 new diagnoses each year [3]. Numerous studies agree that obesity [4], diabetes [5], low physical activity, nulliparity [6], and hyperestrogenism, related or secondary to therapies with tamoxifen [7], are among the main risk factors for endometrial cancer. To date, scientific research is focusing on the search for features of the endometrium that may explain a further predisposition to the onset of endometrial cancer, aimed at improving knowledge of the pathogenetic factors of this disease and the introduction of new preventive therapeutic strategies or early diagnosis. In the last decade, more attention has been paid to the study of the uterine microbiome, thanks also to the advent of new technologies such as new-generation sequencing (NGS), which claims that its composition varies in different pathologies [8]. In past years, it was, in fact, commonly thought that the uterus was a sterile organ [9]. After the first investigations, it was discovered that the endometrium is colonized by its own microbiota. Studies of the vaginal microbiome found its modification in patients suffering from infertility, recurrent miscarriages, as well as cervical carcinomas [10]. The composition of the cervicovaginal microbiome in healthy women with endometrial cancer was subsequently investigated, highlighting the presence of Lactobacillus iners more frequently in healthy patients, and Mobiluncus curtisii and Dialister pneumosintes in patients diagnosed with endometrial cancer. Such bacterial strains appear to play an important role in carcinogenesis, influencing inflammatory responses and increasing the production of pro-inflammatory cytokines [11].

According to recent studies, the occurrence of cellular atypia or malignant transformation may occur under the influence of the proinflammatory microenvironment, especially in inflammatory cells, which provide a favorable environment for neovascularization and the presence of mutations in tumor suppressor proteins or oncoproteins, leading to an increase in cell proliferation and tumor growth [12].

However, to date, studies on the characterization of the microbiome of the endometrium in patients suffering from endometrial carcinoma have detected non-homogeneous microbial colonizations, often due to the smallness or heterogeneity of the sample of women analyzed, or to difficulties in the proper collection of samples, which are often contaminated during collection by cervicovaginal flora.

The aim of our review is to examine in detail the results of the literature on the endometrial microbiota in patients with endometrial cancer and to investigate its role. Considering that endometrial sampling is the method that, more than anything else, exposes studies to bias and contamination by cervicovaginal flora, we selected only the studies in which the endometrial sample was taken after hysterectomy. This is probably the best method of collection considering the low possibility of contamination by vaginal, cervical, and intestinal flora.

## 2. Materials and Methods

We performed our research on MEDLINE (PubMed), Web of Science, and Scopus databases. We searched up to December 2023 and considered manuscripts published from 2000. Only articles in English were included in the search. The research strategy adopted included different combinations of the following terms: (Endometrial Cancer) AND (Microbiota) AND (Microbiome) AND (Endometrial Microbiota).

For the selection of papers, we included only original articles that focused on the study of endometrial microbiota in women with endometrial cancer. We examined in our review the number of patients involved in the study, their hormonal status (pre- or post-menopause), and the bacteria that were identified. We included only studies in which the endometrial sample was collected post-hysterectomy.

We excluded from the review studies that did not report specific information on the bacterial population of endometrial microbiota or that reported only information on vaginal and cervical microbiota. Furthermore, we excluded studies in which the collection of the endometrial microbiota was unclear or collected through the vagina or cervical canal. Articles not relevant to the topic were also excluded.

**Figure 1:**
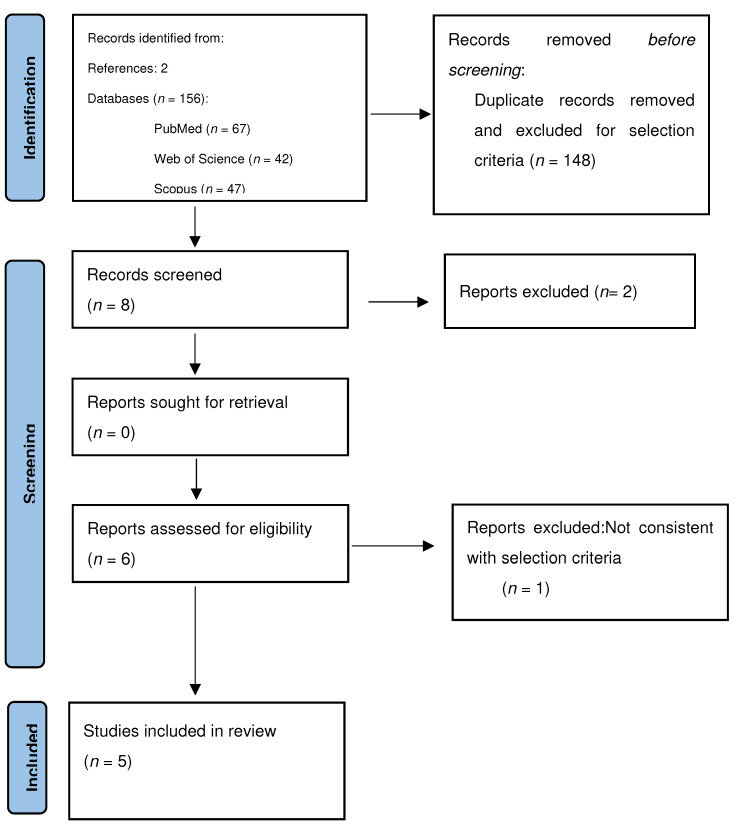
The PRISMA flow diagram of the selection process.

All studies identified were examined for year, citation, title, authors, abstract, and full text. Duplicates were identified through manual screening performed by one researcher and then removed. PRISMA guidelines were followed [13]. The PRISMA flow diagram of the selection process is provided in Figure 1. For the eligibility process, two authors (G.S. and A.D.) independently screened the title and abstracts of all non-duplicated papers and excluded those not pertinent to the topic. The same two authors independently reviewed the full texts of papers that passed the first screening and identified those to be included in the review. Discrepancies were resolved by consensus among the authors. Two manuscripts were detected through the references of the works that had been identified with the research on PubMed and Scopus. The methodological quality of the included studies was assessed using the Joanna Briggs Institute (JBI) Critical Appraisal Checklist for case reports (Table S1 Supplementary).

## 3. Results

We identified 156 manuscripts. Records identified through database searching were 154 (*n* = 67 from Pubmed MEDLINE; *n* = 47 from Scopus; *n* = 42 from Web of Science). Two manuscripts were detected through the references of the works that we recovered from our research on MEDLINE, Web of Science, and Scopus. Records excluded for selection criteria and duplicates were *n* = 151. One manuscript was excluded as it took into consideration only the vagino-cervical microbiome and the intestinal one. Two manuscripts were excluded due to the type of collection of endometrial samples that were at risk of contamination by cervicovaginal flora. At the end of our research, we included in our review five manuscripts, and the total number of patients involved was 190 (Table 1). Four studies considered only post-menopausal patients while one study considered both pre- and post-menopausal patients. In all studies, the microbiota analysis was derived from a post-hysterectomy biopsy.

The microbiota in the endometrial cancer sampling was represented by Bacteroidetes in four of the five manuscripts selected. Bacteroidetes are represented especially by Flavobacterium and Porphyromonas (Gram-negative, anaerobic bacteria). Also, Actinobacteria are reported as the most represented bacteria in patients with endometrial cancer in three of the five manuscripts, with the Atopobium (Gram-positive, anaerobic bacteria) as the most represented species. Also, Firmicutes and Proteobacteria are reported in three of the five manuscripts selected, but with different species present in the three manuscripts. For the Proteobacteria phylum, the most represented bacteria are Pseudomonas, reported in two manuscripts. For this reason, we can state that the isolated bacteria are both Gram-positive and Gram-negative, but predominantly anaerobic.

## 4. Discussion

The results of the literature suggest the presence of a microbial community in the endometrium of healthy women [19,20], but how to definitively confirm its presence and the method for studying its composition are still controversial. The study of the uterine microbiome is difficult considering the constant hormonal changes and the cyclical nature of the menstrual flow, the difficulty of obtaining uterine samples without contaminating the sample with vaginal, cervical, and intestinal bacteria, and the high contamination risk during sample processing. The uterine sample requires invasive methods, and even when a biopsy is performed using explorative techniques that bypass the uterine cervix, women who undergo these procedures are very often already suffering from some pathology or are in peri- or post-menopause, which represents a bias condition [21]. Furthermore, the NGS-based studies in the literature are concentrated on detecting microbial DeoxyriboNucleic Acid (DNA) sequences, but the presence of a microbial DNA does not ensure the presence of a live bacteria. In addition to the type of microbiota, it would be important to understand if and how it could influence the development of endometrial cancer, or whether the change in the microbiota is only a consequence of the presence of the carcinoma and therefore means the study of the microbiota can only be useful for diagnostic purposes. However, regarding the development of colorectal cancer, the literature seems to indicate an increasingly relevant role of the microbiota in terms of stimulation for the immune system and prevention of intestinal dysbiosis which represents a risk factor for colon rectal cancer [22]. Considering the close correlation between the intestinal, vaginal, and uterine microbiota, it is possible to deduce that the endometrial microbiota also plays a role in the development of endometrial cancer and precancerous lesions (endometrial hyperplasia).

According to the studies in the literature, the action of the microbiota on endometrial cancer appears to be multifactorial. The microbiota appears to influence both tumor stroma and cancer cell signaling pathways [23].

Walther-Antonio et al.’s pioneering study reported differences in the composition of microbiota in the upper and lower segments of the female genitalia in women undergoing hysterectomy for endometrial cancer, endometrial hyperplasia, and benign pathology. His study and others have shown the existence of differences in the endometrial microflora in benign conditions compared to endometrial tumors, proposing an effect of the microflora in the early stages of cellular transformation and in the progression of the pathology [14,24]. In the same study, sequencing the 16S rDNA V3-V5 region in endometrial cancer patients underlined the important role of Bacteroides and Faecalibacterium. This confirms that bacteria of the genus Bacteroides are the prevalent taxa of the uterus [14]. From our review, the Bacteroides genus seems to be dominant in patients with endometrial carcinoma, also isolating Porphyromonas as a bacterium. Porphyromonas sp. have been isolated intracellularly in other studies that report the possibility for these bacteria to alter the cellular regulatory processes, leading to the process of carcinogenesis [24]. Further studies showed that the presence of uterine microbiota of Porphyromonas somerae in obese menopausal patients was highly predictive of the presence of uterine cancer [25]. Considering the role of Bacteroidetes, previous studies have shown that endometrial cancer seems to be related to an altered expression of genes associated with fibrin breakdown. In particular, the study of Li et al. showed that the altered expression was related to an increase in the presence of *Prevotella* sp., a bacteria overexpressed also in the study of Wang et al. [18], indicating a possible role of Prevotella in the process of host fibrin breakdown leading to endometrial cancer [26]. In the manuscript of Wanting Lu et al., Micrococcus seems to have a direct correlation with inflammatory cytokines, and these could be involved in the development of EC. In fact, cytokines have the ability to modify the local microenvironment and could be implicated in gynecologic cancer development through increased angiogenesis, cellular proliferation, and modification of the local immune response [15]. However, the study of Wanting Lu et al. disagrees with the work of Walter-Antonio et al. on microbial diversity. In the literature, reduced microbial diversity is often associated with chronic diseases such as diabetes, IBDs, and cancer [27] and in the study of Wanting Lu the microbial diversity is reduced in patients with EC, while the study of Walther-Antonio reported that diversity was increased in the EC group [14]. However, the limit of this study is represented by the small sample size (31 subjects), and this could have influenced the results. Furthermore, other factors like systemic inflammation, infection, immune response, diet, and lifestyle could influence microbial diversity [14,28].

Also, the study of Gressel et al. demonstrated a significant reduction in microbial diversity, especially in patients with uterine serous cancers compared to endometrioid uterine cancer and controls, as if with the worsening of the pathology and histotype, there is a gradual reduction in uterine bacterial diversity. Furthermore, in addition to a reduction in uterine bacterial diversity, they have demonstrated a significant correlation between lower vaginal Lactobacillus and elevated uterine Pseudomonas associated with uterine serous cancers. The presence of Pseudomonas also seems to be related to endometrial cancer in the work of Walther-Antonio et al. [14].

In the literature, many studies in which endometrial samples were collected transcervically have shown the dominance of lactobacilli within the endometrial microflora [29]. This type of result may have been altered by the fact that Lactobacillus is predominant in the vaginal microbiota, and if the endometrial sample collection is contaminated from the cervical or vaginal canal, it will give a higher quantity of Lactobacillus than the real amount. Some studies have demonstrated that the presence of Lactobacillus has a positive association with genitourinary health [30]. Lactobacillus may be acting to limit carcinogenesis by reducing local inflammation and modulating cytokine activity [16].

The study of Hawkins et al. [17] is important considering that they have identified the Actinobacteria, Bacteroidetes, Firmicutes, OD1, and Proteobacteria phyla in both the benign and malignant uterine tissue specimens, similar to what has previously been shown in other studies. Furthermore, as in the study of Walther-Antonio et al., they found a greater microbial diversity and a higher abundance of microbes in eCs when compared to the benign uterus. In this study, therefore, rather than the presence of specific bacteria, it seems to be the quantity of the same that is different in healthy patients compared to patients with endometrial cancer. Significant differences based on obesity status were seen at the phylum level, with a higher microbial diversity in the eCs in obese compared to non-obese White women. In their study, they assessed differences based on race, demonstrating that microbial diversity was higher in the eCs from Black versus White women. Hawkins et al. showed that L. acidophilus was higher in the eCs of Black women, even though a previous study demonstrated that White and Asian women have more Lactobacillus dominant vaginal communities than Hispanic and Black women, showing that the role of the Lactobacillus community on uterine health and disparities in its presence is more complex [17]. The study of Wang et al. [18] underlined some connections between endometrial microbiota shift and EC progression. The concept of endometrial microbiota shift had also been suggested in the work of Gressel et al. but only from the point of view of the reduction in microbial diversity. In fact, confirming the results of previous studies [14,31], Wang et al. found an increased richness of the genera Prevotella, Atopobium, Anaerococcus, Dialister, Porphyromonas, and Peptoniphilus in the EC endometrium. Overall, this study indicates that EC and adjacent EC-healthy endometrium in post-menopausal individuals have significantly different microbiota, and Gardnerella, Atopobium, Fastidiosipila, and Sneathia were positively correlated with the stage of the tumor [18]. The intuition of the microbiota shift is probably the most important one, but more data is needed to better understand how the microbiota influences the development of endometrial cancer or if its change is only a consequence of the progression of the tumor itself. If the microbiota shift is confirmed, new therapeutic possibilities would be possible by acting on the composition of the microbiota to influence the development of the tumor. In fact, if the quantity of a specific bacterium is important rather than its presence (according to some studies we have taken into consideration), and if an increase in certain bacteria leads to a gradual shift from a benign picture to one of malignancy, future studies may focus on the possibility of modifying the microbiota in cases in which the endometrium manifests precancerous lesions (ex. atypical hyperplasia) so as to inhibit the processes that lead to cancer.

Studies of this type are already underway for other pathologies related to the lower genital tract [32,33].

A strength of our study is represented by the long period of time considered for our research on databases. We have included only one study in which the endometrial sample was collected post-hysterectomy to reduce the possibility of contamination by the vaginal, cervical, and intestinal flora. We have used the JBI Critical Appraisal Checklist for cohort studies to assess the trustworthiness, relevance, and results of the published papers.

The main limitation of this review is the inclusion of only five studies and 190 patients among the papers selected. Furthermore, almost all patients were in the post-menopause period. This is due to the few studies present in the literature on the subject, on small samples of patients and in which the collection of endometrial samples is exposed to the risk of contamination.

## 5. Conclusions

From our review, it emerged that Bacteroidetes, Actinobacteria, Firmicutes, and Proteobacteria are the most represented bacteria in patients with endometrial cancer. They are both Gram-positive and Gram-negative, but predominantly anaerobic bacteria. The mechanism by which they influence the development of endometrial carcinoma remains unclear but is probably multifactorial. Even if some studies are divergent, reduced microbial diversity and the presence of specific bacteria are often associated with endometrial cancer. Some studies have introduced the concept of endometrial microbiota shift and EC progression, with changes in the microbiota and a reduction in microbial diversity along with the development of endometrial cancer and worsening of the histotype. Further work on larger population samples and both healthy women and those affected by endometrial carcinoma is needed to understand how the endometrial microbiota changes and influences the development of tumors. Furthermore, future studies will have to focus on the possibility of modifying the microbiota to inhibit the processes that lead to cancer.

## Figures and Tables

**Table 1:** Manuscripts included in the review.

Authors	N Patients with EC	Pre/Post-Menopause	Microbiota in EC—Phylum (Genus/Species)	Sampling Type
**Walther-Antonio MRS et al. 2016 [14]**	17	Post-menopause	Firmicutes (Anaerostipes, ph2, Dialister, Peptoniphilus, 1–68, Ruminococcus, Anaerotruncus), Spirochaetes (Treponema), Actinobacteria (Atopobium), Bacteroidetes (Bacteroides, Porphyromonas), Proteobacteria (Arthrospira)	Post-hysterectomy biopsy
**Lu W et al. 2020 [15]**	25	Pre- and post-menopause	Actinobacteria (Micrococcus), Firmicutes (Pseudoramibacter, Eubacterium, Megamonas), Proteobacteria (Rhodobacter, Vogesella, Bilophila, Rheinheimera)	Post-hysterectomy biopsy
**Gressel GM et al. 2021 [16]**	25	Post-menopause	Bacteroidetes (Flavobacterium)	Post-hysterectomy biopsy
**Hawkins GM et al. 2022 [17]**	95	Post-menopause	Bacteroidetes (Flavobacterium), Pseudomonadota (Pelomonas, Hyphomicrobium, Bradyrhizobium), Proteobacteria (Pseudomonas, Acidovorax)	Post-hysterectomy biopsy
**Wang L et al. 2022 [18]**	28	Post-menopause	Bacteroidetes (Prevotella, Porphyromonas), Actinobacteria (Atopobium), Firmicutes (Anaerococcus, Dialister, Peptoniphilus)	Post-hysterectomy biopsy

## Data Availability

The authors confirm that the data supporting the findings of this study are available within the article.

## Supplementary Materials

The following supporting information can be downloaded at: https://www.mdpi.com/article/10.3390/jcm13237135/s1, Table S1: JBI checklist for case series.
