# Supplementary material for: The Role of the Endometrial Microbiota in Endometrial Cancer: A Systematic Review of the Literature
Source: J Clin Med. 2024 Nov 25;13(23):7135. doi: 10.3390/jcm13237135 (PMC11642298; doi:10.3390/jcm13237135)
Supplement: Supplementary file 1 [file jcm-13-07135-s001.zip › jcm-3244965-supplementary (1).pdf]

Table S1: JBI checklist for case series.

Author, Year	D1	D2	D3	D4	D5	D6	D7	D8	D9	D10
Walther-António et al 2016	Yes	Yes	Yes	Yes	No	Yes	No	No	Yes	Yes
Wanting Lu et al 2020	Yes	Yes	Yes	Yes	Yes	Unclear	No	Unclear	Unclear	Yes
Gressel et al 2021	Yes	Yes	Yes	Yes	Yes	Yes	Yes	Yes	Yes	Yes
Hawkins et al 2022	Yes	Yes	Yes	Yes	Yes	Yes	Unclear	Yes	Yes	Yes
Wang et al 2022	Yes	Yes	Yes	Yes	Yes	Yes	Unclear	Yes	Yes	Yes
